# A Prediction Model for Acute Kidney Injury After Pericardiectomy: An Observational Study

**DOI:** 10.3389/fcvm.2022.790044

**Published:** 2022-02-11

**Authors:** Jin Wang, Chunhua Yu, Yuelun Zhang, Yuguang Huang

**Affiliations:** ^1^Department of Anesthesiology, Peking Union Medical College Hospital, Chinese Academy of Medical Sciences, Beijing, China; ^2^Department of Biostatistics, Peking Union Medical College Hospital, Chinese Academy of Medical Sciences, Beijing, China

**Keywords:** acute kidney injury, constrictive pericarditis, pericardiectomy, prediction model, KDIGO (Kidney Disease Improving Global Outcomes), nomogram

## Abstract

**Objectives:**

Acute kidney injury is a common complication after pericardiectomy for constrictive pericarditis, which predisposes patients to worse outcomes and high medical costs. We aimed to investigate potential risk factors and consequences and establish a prediction model.

**Methods:**

We selected patients with constrictive pericarditis receiving isolated pericardiectomy from January 2013 to January 2021. Patients receiving concomittant surgery or repeat percardiectomy, as well as end-stage of renal disease were excluded. Acute kidney injury was diagnosed and classified according to the KDIGO criteria. Clinical features were compared between patients with and without postoperative acute kidney injury. A prediction model was established based on multivariable regression analysis.

**Results:**

Among two hundred and eleven patients, ninety-five (45.0%) developed postoperative acute kidney injury, with fourty-three (45.3%), twenty-eight (29.5%), and twenty-four (25.3%) in mild, moderate and severe stages, respectively. Twenty-nine (13.7%) patients received hemofiltration. Nine (4.3%) patients died perioperatively and were all in the acute kidney injury (9.5%) group. Eleven (5.2%) patients were considered to have chronic renal dysfunction states at the 6-month postoperative follow-up, and eight (72.7%) of them experienced moderate to severe stages of postoperative acute kidney injury. Univariable analysis showed that patients with acute kidney injury were older (difference 8 years, *P* < 0.001); had higher body mass index (difference 1.68 kg·m^−2^, *P* = 0.002); rates of smoking (*OR* = 2, *P* = 0.020), hypertension (*OR* = 2.83, *P* = 0.004), and renal dysfunction (*OR* = 3.58, *P* = 0.002); higher central venous pressure (difference 3 cm H_2_O, *P* < 0.001); and lower cardiac index (difference −0.23 L·min^−1^·m^−2^, *P* < 0.001) than patients without acute kidney injury. Multivariable regression analysis showed that advanced age (OR 1.03, *P* = 0.003), high body mass index (OR 1.10, *P* = 0.024), preoperative atrial arrhythmia (OR 3.12, *P* = 0.041), renal dysfunction (OR 2.70 *P* = 0.043), high central venous pressure (OR 1.12, *P* = 0.002), and low cardiac index (OR 0.36, *P* = 0.009) were associated with a high risk of postoperative acute kidney injury. A nomogram was established based on the regression results. The model showed good model fitness (Hosmer-Lemeshow test *P* = 0.881), with an area under the curve value of 0.78 (95% CI: 0.71, 0.84, *P* < 0.001).

**Conclusion:**

The prediction model may help with early recognition, management, and reduction of acute kidney injury after pericardiectomy.

## Introduction

Pericardiectomy is the most common and only definitive treatment for constrictive pericarditis (1). Patients with constrictive pericarditis have been reported to have a high incidence of both pre- and postoperative renal dysfunction, which predisposes them to poor outcomes and high medical costs (2–4). Previous studies have reported that the incidence of postoperative renal failure and dialysis after isolated or committant pericardiectomy were approximately 3–15 and 3–30%, respectively (4–7). However, the definition of renal failure was unclear, and no details of the causes or potentional risk factors were investigated. Predictive analytics have been widely used in the surgery literature for postoperative complications (8). To the best of our knowledge, we failed to identify any study exploring the preoperative predictors of acute kidney injury after pericardiectomy.

This single-center observational study aimed to investigate the potential predictors of acute kidney injury after pericardiectomy in patients with constrictive pericarditis. Perioperative clinical features were compared between patients with and without acute kidney injury, and a prediction model was established based on the comparison results.

## Materials and Methods

### Participants and Setting

This study was conducted in a tertiary hospital in Beijing, China. The hospital's institutional review board approved the study (No. S-K948), and patient informed consent was waived. The following inclusion criteria were used: patients who received isolated pericardiectomies for constrictive pericarditis. The following exclusion criteria were used: patients who received repeat pericardiectomy or concomitant cardiac procedures, such as coronary artery bypass grafting and valve surgeries, and patients already have end-stages of renal disease.

### Variables and Measurement

The essential variables that were collected included preoperative comorbidities, etiologies, distinctive clinical manifestations, and hemodynamic parameters, and postoperative complications and outcomes.

#### Preoperative Data

Preoperative comorbidities were collected by checking the past medical histories and preoperative examination results of the patients. The major comorbidities included hypertension, diabetes mellitus, coronary artery disease, myocardial infarction, cerebral infarction, and renal disease. Etiologies of the studied population included infection, postsurgery, postacute myocardial infarction (AMI), radiation and idiopathic causes. Infectious pericarditis was divided into tuberculosis (TB) and non-TB infection, based on pathological results, anti-TB treatment effects and a guideline-recommended diagnostic flowchart (1). Postsurgical constrictive pericarditis was considered if the patient had a history of prior mediastinal surgery. Post-AMI constrictive pericarditis was considered if the patient had post-AMI pericarditis syndrome. Postradiation constrictive pericarditis was considered if the patient had a history of prior mediastinal radiation. Patients were classified into the idiopathic group if none of the above etiologies were qualified (9, 10).

The distinctive clinical manifestations that were evaluated included signs of fluid overload and major organ dysfunction, including cardiac, hepatic, and renal dysfunctions. Peripheral edema was determined by physical examination. Pleural effusion, ascites, pericardial calcification and hepatomegaly were determined from the ultrasound and computed topography imaging results. Atrial arrhythmia, including atrial flutter and fibrillation, was determined from the medical history and electrocardiogram. Biochemical disturbances including moderate to severe hypokalemia (serum potassium <3 mmol·L^−1^), moderate to severe hyponatremia (serum sodium <130 mmol·L^−1^), hypoalbuminemia (albumin <35 g∙L^−1^), coagulopathy and renal dysfunction (serum creatinine >106 μmol·L^−1^ in males and >97 μmol·L^−1^ in females), were determined from the blood test results. The test results of patients with prior uses of anticoagulation medication were excluded from the coagulopathy evaluation.The hemodynamic parameters collected included central venous pressure and cardiac index. Central venous pressure was measured via a central venous catheter, and the cardiac index was measured by using the thermodilution method with a pulse index continuous cardiac output device.

#### Outcomes and Definition

All of the patients underwent total pericardiectomy with median sternotomy under general anesthesia. The surgeons removed as much of the constricting pericardium as possible, including all of the anterior and diaphragmatic pericardium (phrenic nerve to phrenic nerve), as well as all of the accessible portion of the pericardium posterior to the left phrenic nerve. Liberation of the right atrium, along with the superior and inferior vena cavae and inferior part of the right ventricle adjacent to the diaphragm, was also performed to the greatest possible extent.

The primary end point was postoperative acute kidney injury, defined and staged according to the Kidney Disease Improving Global Outcomes (KDIGO) criteria: (1) mild stage: serum creatinine increased to 1.5–1.9 times baseline or ≥0.3 mg·dl^−1^ or urine output <0.5 ml·kg^−1^·h^−1^ for 6–12 h; (2) moderate stage: serum creatinine increased to 2.0–2.9 times baseline, or urine output <0.5 ml·kg^−1^·h^−1^ for ≥12 h; (3) severe stage: serum creatinine increased to 3.0 times baseline or ≥0.4 mg·dl^−1^; initiation of renal replacement therapy; in patients <18 years old, a derease in eGFR to <35 ml·min^−1^ per 1.73 m^2^, or <0.3 ml·kg^−1^·h^−1^ for ≥24 h or anuria for ≥12 h (11, 12). Baseline serum creatinine levels were tested on the day before surgery. The second end points were postoperative mortality and chronic renal dysfunction. Mortality was defined as any postoperative death that occurred during the same hospital admission or within 30 days after discharge. Chronic renal dysfunction was determined according to the KDIGO criteria at the 6-month postoperative follow-up (13).

Other postoperative complications that were recorded included low cardiac output, tachyarrhythmia, delirium, and the use of mechanical circulatory support devices. Low cardiac output was determined if patients required more than one inotrope to maintain enough organ perfusion. Tachyarrhythmia included new onset atrial fibrillation, atrial fibrillation with a rapid ventricular rate and supraventricular and ventricular tachycardia in the absence of electrolyte abnormalities. Delirium was evaluated by using the Confusion Assessment Method-Intensive Care Unit score every twelve hours and at times when delirium was suspected (14, 15). Uses of mechanical circulatory support devices, including IABP, ECMO and hemofiltration, were also recorded. Other outcomes included ventilator hours and lengths of intensive care unit (ICU) and hospital stays. Follow-up was performed until 6 months after the operation. Patient status was determined from either a clinical visit or a telephone call.

A dedicated data coordinating team performed all data management. Prespecified demographics and clinical and laboratory information were obtained from hospital charts that were reviewed by independent research personnel who were unaware of the objectives of the study, and accumulated data were then entered prospectively into the database.

## Statistical Analysis

Statistical analyses were performed with SPSS 24.0.0.0 software (IBM Corp) and R 4.1.2 packages. Normality was tested with a Q-Q plot. Continuous variables with a normal distribution are expressed as the mean ± standard deviation; additionally, continuous variables with a non-normal distribution are expressed as medians (quartile), and categorical variables are expressed as case numbers and percentages. The independent *t* test was performed to analyze the continuous variables with a normal distribution. The Mann-Whitney *U* tests were used for the analysis of continuous variables with a non-parametric distribution. The chi-square test was used to evaluate categorical data when the expected cell counts were >5; otherwise, Fisher's exact test was used.

Preoperative variables with a *P* < 0.1 or considered to be clinically related to acute kidney injury, including age, BMI, hypertension, smoking, atrial arrhythmia, renal dysfunction, central venous pressure, and cardiac index, were selected for multivariable logistic regression using forward stepwise analysis and are shown in a coefficient plot. A nomogram was established based on the regression results. Model performance was evaluated using the same dataset as was used for model training due to the limited number of cases. Calibration was assessed with the Hosmer-Lemeshow goodness-of-fit statistic and calibration plot. Model discrimination was evaluated using the area under the receiver operating characteristic curve. All of the tests were two-tailed, and a *P* < 0.05 was considered to be statistically significant.

## Results

### Participants and Descriptive Data

From January 2013 to January 2021, a total of 253 patients underwent pericardiectomy for constrictive pericarditis in the studied hospital. Nineteen combined operations were excluded from the study, including ten coronary artery bypass grafting surgeries, six valve surgeries, two tumor resections, and one ventricular aneurysm repair. Twenty-three patients with lost medical data were also excluded from the study. A total of 211 patients with constrictive pericarditis receiving pericardiectomy were included in the final analysis.

### Main Results

Among the 211 patients, ninety-five (45.0%) of them developed postoperative acute kidney injury, with fourty-three (45.3%), twenty-eight (29.5%), and twenty-four (25.3%) patients in mild, moderate and severe stages, respectively. All clinical data were summarized and compared between patients with (AKI group) and without (non-AKI group) postoperative acute kidney injury (Tables 1–**4**).

**Table 1:** Baseline characteristics of patients with and without acute kidney injury after pericardiectomy for constrictive pericarditis.

	**Total** **(***N*** = 211)**	**AKI** **(***N =*** 95)**	**Non-AKI** **(***N =*** 116)**	**OR/Difference (95%CI)**	***P***
Male sex (*n*, %)	155, 73.5%	75, 78.9%	80, 69.0%	1.69 (0.90, 3.17)	0.102
Age (years old)	46 ± 16	50 ± 15	42 ± 17	8 (4, 12)	<0.001^*^
Body mass index (kg·m^−2^)	22.30 + 4.01	23.22 ± 4.08	21.55 ± 3.80	1.68 (0.61, 2.75)	0.002^*^
Smoking (*n*, %)	67, 31.8%	38, 40%	29, 25.0%	2.00 (1.11, 3.60)	0.020^*^
Hypertension (*n*, %)	38, 18.0%	25, 26.3%	13, 11.2%	2.83 (1.36, 5.91)	0.004^*^
Diabetes mellitus (*n*, %)	20, 9.5%	12, 12.6%	8, 6.9%	1.95 (0.76, 4.99)	0.157
Coronary artery disease (*n*, %)	28, 13.3%	13, 13.7%	15, 12.9%	1.07 (0.48, 2.37)	0.873
Myocardial infarction (*n*, %)	4, 1.9%	2, 2.1%	2, 1.7%	1.23 (0.17, 8.87)	0.999
Cerebral infarction (*n*, %)	9, 4.3%	5, 5.3%	4, 3.4%	1.56 (0.41, 5.96)	0.734
Kidney disease history (*n*, %)	5, 2.4%	2, 2.2%	3, 2.6%	0.81 (0.13, 4.95)	0.999

* *Significant difference; AKI, acute kidney injury; OR, odds ratio; CI, confidential interval*.

For baseline characteristics, the AKI group was older (difference 8 years old, 95% CI 4–12 years old, *P* < 0.001) and had a higher BMI (difference 1.68 kg·m^−2^, 95% CI 0.61–2.75 kg·m^−2^, *P* = 0.002) than the non-AKI group. The AKI group had higher rates of smoking (OR 2.00, 95% CI 1.11–3.60, *P* = 0.020) and hypertension (OR 2.83, 95% CI 1.36–5.91, *P* = 0.004) than the non-AKI group. No differences in other comorbidities were observed between the AKI and non-AKI group (Table 1). Etiologies of constrictive pericarditis included idiopathic (81, 38.4%), infection (121, 57.3%), prior cardiac surgery (1, 0.5%), post myocardial infarction (2, 0.9%), and radiation (5, 2.4%). No differences in etiologies were detected between the AKI and non-AKI groups (Table 2).

**Table 2:** Etiologies of patients with and without acute kidney injury after pericardiectomy for constrictive pericarditis.

**Etiologies** **(***n***, %)**	**Total** **(***N =*** 211)**	**AKI** **(***N =*** 95)**	**Non-AKI** **(***N =*** 116)**	**Odds ratio (95%CI)**	***P***
Idiopathic	81, 38.4%	35, 36.8%	46, 39.7%	0.89 (0.51, 1.55)	0.676
Tuberculosis	110, 52.1%	47, 49.5%	63, 54.3%	0.83 (0.48, 1.42)	0.484
Non-TB infection	11, 5.2%	7, 7.4%	4, 3.4%	2.23 (0.63, 7.85)	0.203
Prior surgery	1, 0.5%	0, 0.0%	1, 0.9%	0.60 (0.05, 6.77)	0.680
Post MI	2, 0.9%	1, 1.1%	1, 0.9%	1.22 (0.08, 19.82)	0.887
Radiation	5, 2.4%	4, 4.3%	1, 0.9%	5.06 (0.56, 46.01)	0.177

*AKI, acute kidney injury; OR, odds ratio; CI, confidential interval; TB, tuberculosis; MI, myocardial infarction*.

Preoperatively, the overall rates of renal dysfunction, atrial arrhythmia, hyponatremia, hypoalbuminemia and pericardial calcification were 14.7, 12.3, 10.0, 48.8, and 21.3%, respectively. A total of thirty-one (14.7%) patients had renal dysfunction before pericardiectomy, and twenty-two (71.0%) of them developed postoperative acute kidney injury. The overall mean central venous pressure was 17 ± 5 cmH_2_O, and the cardiac index was 1.87 ± 0.45 L·min^−1^·m^−2^. For the between group comparison, the AKI group had higher rates of renal dysfunction (OR: 3.58, 95% CI 1.56–8.22, *P* = 0.002), higher central venous pressure (difference 3 cmH_2_O, 95% CI 1–4 cmH_2_O, *P* < 0.001) and lower cardiac index (difference −0.23 L·min^−1^·m^−2^, 95% CI −0.34 to −0.11 L·min^−1^·m^−2^, *P* < 0.001) than the non-AKI group (Table 3).

**Table 3:** Preoperative features of patients with and without acute kidney injury after pericardiectomy for constrictive pericarditis.

**Clinical features** **(***n***, %)**	**Total** **(***N =*** 211)**	**AKI** **(***N =*** 95)**	**Non-AKI** **(***N =*** 116)**	**OR/Difference (95%CI)**	***P***
Pleural effusion	177, 83.9%	84, 88.4%	93, 80.2%	1.89 (0.87, 4.11)	0.105
Ascites	154, 73.0%	73, 76.8%	81, 69.8%	1.43 (0.77, 2.67)	0.254
Peripheral edema	182, 86.3%	85, 89.5%	97, 83.6%	1.67 (0.73, 3.78)	0.219
Hepatomegaly	115, 54.5%	53, 55.8%	62, 53.4%	1.10 (0.64, 1.90)	0.734
Renal dysfunction	31, 14.7%	22, 23.2%	9, 7.8%	3.58 (1.56, 8.22)	0.002^*^
Coagulopathy	154.73.0%	73, 76.8%	81, 69.8%	1.43 (0.77, 2.67)	0.254
Hyponatremia	21, 10.0%	13, 13.7%	8, 6.9%	2.14 (0.85, 5.41)	0.101
Hypoalkemia	22, 10.4%	9, 9.5%	13, 11.2%	0.83 (0.34, 2.03)	0.682
Hypoalbuminemia	103, 48.8%	52, 54.7%	51, 44%	1.54 (0.89, 2.66)	0.119
Pericardial calcification	45, 21.3%	21, 22.1%	24, 20.7%	1.09 (0.56, 2.11)	0.803
Atrial arrhythmia	26, 12.3%	16, 16.8%	10, 8.6&	2.15 (0.93, 4.98)	0.071
CVP (cmH_2_O)	17 ± 5	19 ± 5	16 ± 4	3 (1,4)	<0.001^*^
CI (L·min^−1^ ·m^−2^)	1.87 ± 0.45	1.75 ± 0.44	1.97 ± 0.43	−0.23 (−0.34, −0.11)	<0.001^*^

* *Significant difference*.

*AKI, acute kidney injury; OR, odds ratio; CI, confidential interval; CVP, central venous pressure; CI, cardiac index*.

Postoperatively, the AKI group had higher rates of complications, including low cardiac output (OR 9.15, 95% CI 4.26–19.62, *P* < 0.001), tachyarrhythmia (OR: 8.61, 95% CI 3.76–19.71, *P* < 0.001), delirium (OR: 12.03,95% CI 3.49–41.53, *P* < 0.001); and use of hemofiltration (OR: 48.06, 95% CI 6.39–361.30, *P* < 0.001), ECMO (OR: 9.10, 95% CI 1.10–75.30, *P* = 0.014), and IABP (OR: 10.52, 95% CI 1.29–85.63 *P* = 0.007) than the non-AKI group. A total of twenty-nine (13.7%) patients received hemofiltration, with twenty-eight (29.5%) patients in the AKI group, and one patient in the non-AKI group. One patient in the non-AKI group started preventive hemofiltration early after pericardiectomy, because the clinicians considered him at high risk of developing low cardiac output due to a dramatic fluid shift back into the intravascular space. The AKI group had longer ventilator hours (median difference 54 h, 95% CI 37–66 h, *P* < 0.001) and lengths of ICU (median difference 3 days, 95% CI 2–4 days, *P* < 0.001) and hospital stays (median difference 7 days, 95% CI 4–10 days, *P* < 0.001) than the non-AKI group (Table 4).

**Table 4:** Postoperative outcomes of patients with and without acute kidney injury after pericardiectomy for constrictive pericarditis.

**Outcomes** **(***n***, %)**	**Total** **(***N =*** 211)**	**AKI** **(***N =*** 95)**	**Non-AKI** **(***N =*** 116)**	**OR/Difference (95%CI)**	***P***
Low cardiac output	54, 25.6	44, 46.3%	10, 8.6%	9.15 (4.26, 19.62)	<0.001^*^
Tachyarrhythmia	45, 21.3%	37, 38.9%	8, 6.9%	8.61 (3.76, 19.71)	<0.001^*^
Hemofiltration	29, 13.7%	28, 29.5%	1, 0.9%	48.06 (6.39, 361.30)	<0.001^*^
Chronic renal dysfunction	11, 5.2%	9, 9.5%	2, 1.7%	5.97 (1.26, 28.31)	0.012^*^
Delirium	26, 12.3%	23, 24.2%	3, 2.6%	12.03 (3.49, 41.53)	<0.001^*^
Diaphragmatic paralysis	7, 3.3%	5, 5.3%	2, 1.7%	3.18 (0.60, 16.70)	0.153
ECMO	6, 2.8%	6, 6.3%	0, 0.0%	9.10 (1.10, 75.30)	0.014^*^
IABP	7, 3.3%	7, 7.4%	0, 0.0%	10.52 (1.29, 85.63)	0.007^*^
Ventilator (hours)	30 (18, 84)	78 (36, 78)	20 (15, 30)	54 (37, 66)	<0.001^*^
ICU stay (days)	3 (2,6)	5 (3, 12)	2 (2,3)	3 (2, 4)	<0.001^*^
Length of stay (days)	24 (17, 34)	28 (28, 41)	21 (15, 28)	7 (4, 10)	<0.001^*^
Mortality (*n*, %)	9, 4.3%	9, 9.5%	0, 0.0%	13.45 (11.69, 107.03)	0.002^*^

* *Significant difference*.

*AKI, acute kidney injury; OR, odds ratio; CI, confidential interval; ECMO, extracorporeal membrane oxygenation; IABP, intra-aortic balloon pump; ICU, intensive care unit*.

Regarding mortality, the AKI group had higher mortality (OR: 13.45, 95% CI 11.69–107.03, *P* = 0.001) than non-AKI group. All deaths (9 patients, 9.5%) occurred in the AKI group. Seven patients died in the hospital due to multiple organ dysfunction. Two patients were self-discharged due to financial reasons, with one patient dying of multiple organ dysfunction within hours and the other patient dying of an unknown cause within days.

At the 6-month postoperative follow up, eleven (5.2%) patients were considered in chronic renal dysfunction states, with nine from the AKI group and two from the non-AKI group (OR: 5.97, 95% CI 1.26–28.31, *P* = 0.012). The two patients in the non-AKI group already had chronic renal dysfunction before pericardiectomy due to a previous history of renal disease. All the clinical features were compared between patients with (CRD group) and without (non-CRD group) postoperative chronic renal dysfunction. Parameters with statistically significant between-group differences are listed in Table 5.

**Table 5:** Comparison of clinical data between patients with and without chronic renal dysfunction at 6 month postoperative follow-up.

**Clinical features**	**CRD**	**Non-CRD**	**Difference/OR (95%CI)**	***P***
	**(***n*** = 11)**	**(***n*** = 191)**		
**Preoperative features (** ***n*** **, %)**				
Smoking	7, 63.6%	57, 29.8%	4.11 (1.16, 14.61)	0.039^*^
Kidney disease history	2,18.2%	3, 1.6%	13.93 (2.06, 94.05)	0.025^*^
Atrial arrhythmia	4, 36.4%	20, 10.5%	4.89 (1.31, 18.16)	0.029^*^
Renal dysfunction	6, 54.5%	23, 12.0%	8.77 (2.48, 31.03)	0.001^*^
Cardiac index (L∙min^−1^ ∙m^−2^)	1.56 ± 0.41	1.90 ± 0.45	−0.33 (−0.61, −0.05)	0.020^*^
**Postoperative outcomes (** ***n*** **, %)**				
Low cardiac output	7, 63.6%	40, 20.9%	6.61 (1.84, 23.69)	0.004^*^
Acute kidney injury	9, 81.8%	77, 40.3%	6.66 (1.40, 31.68)	0.010^*^
Tachyarrhythmia	8, 72.7%	31, 16.2%	13.76 (3.46, 54.79)	<0.001^*^
Hemofiltration	6, 54.5%	20, 10.5%	10.26 (2.87, 36.68)	0.001^*^
Ventilator (hours)	93 (66, 214)	26 (17,74)	64 (8, 97)	0.016^*^

*CRD, Chronic renal dysfunction*.

* *Significant difference*.

Preoperatively, the CRD group had higher rates of smoking (OR: 4.11, 95% CI 1.16–14.61, *P* = 0.039), kidney disease history (OR: 13.93, 95% CI 2.06–94.05, *P* = 0.025), atrial arrhythmia (OR: 4.89, 95% CI 1.31–18.16, *P* = 0.029), and renal dysfunction (OR: 8.77, 95% CI 2.48–31.03, *P* = 0.001) than the non-CRD group. The cardiac index was lower in the CRD group than in the non-CRD group (difference −0.33 L·min^−1^ ·m^−2^, 95% CI −0.61 to −0.05 L·min^−1^ ·m^−2^, *P* = 0.020). Postoperatively, the CRD group had higher rates of low cardiac ouput (OR: 6.61, 95% CI 1.84–23.69, *P* = 0.004), acute kidney injury (OR: 6.66, 95% CI 1.40–31.68, *P* = 0.010), tachyarrhythmia (OR: 13.76, 95% CI 3.46–54.79, *P* < 0.001), and use of hemofilatration (OR: 10.26, 95% CI 2.87–36.68, *P* = 0.001) than the non-CRD group. The ventilator duration was longer (median difference 64 h, 95%CI 8–97 h, *P* = 0.016) in the CRD group than in the non-CRD group. The other clinical data were similar between the CRD and non-CRD groups. These results should be carefully interpreted due to the limited case numbers in the CRD group.

**Figure 1:**
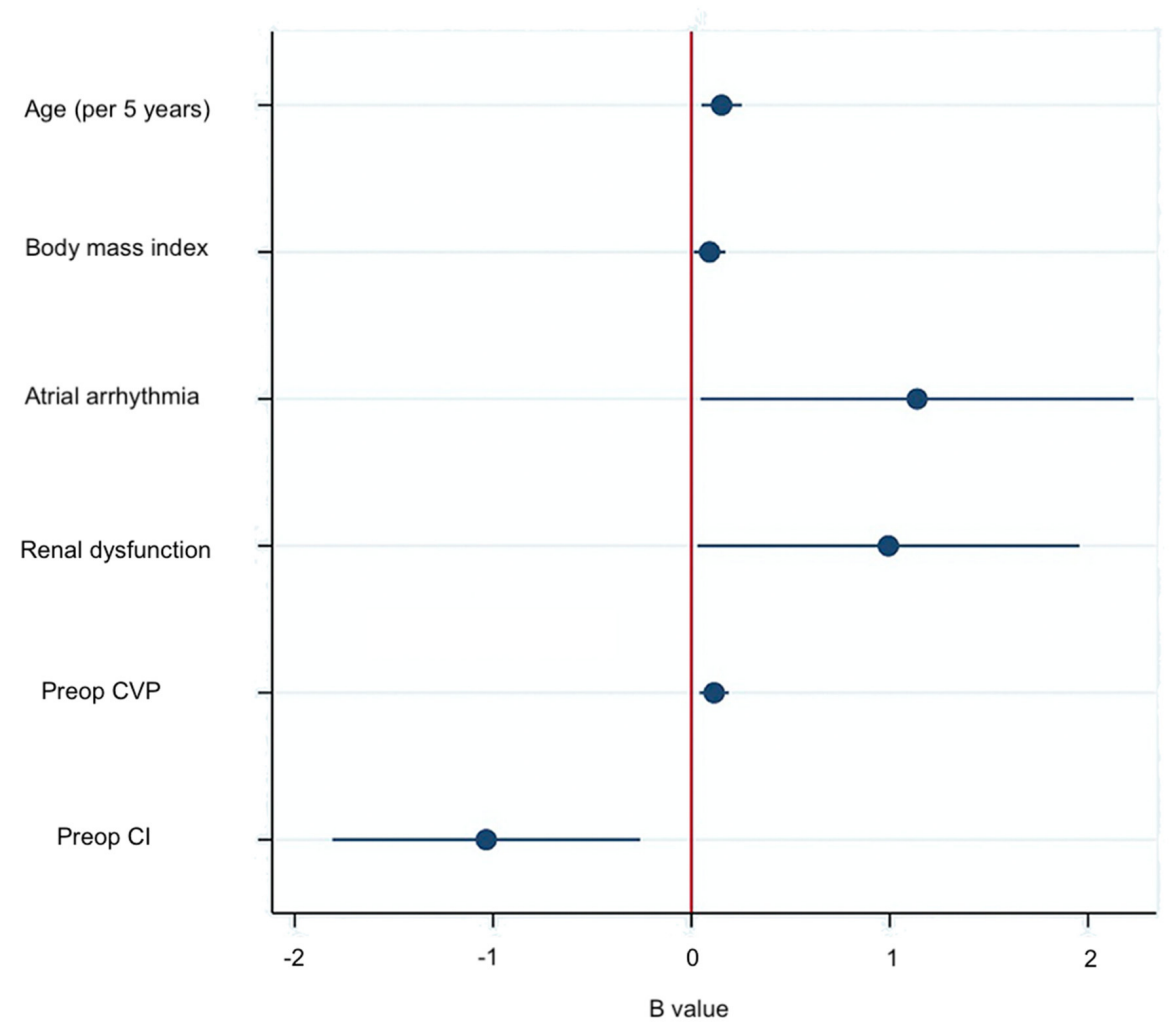
Predictors of acute kidney injury after pericardiectomy.

**Figure 2:**
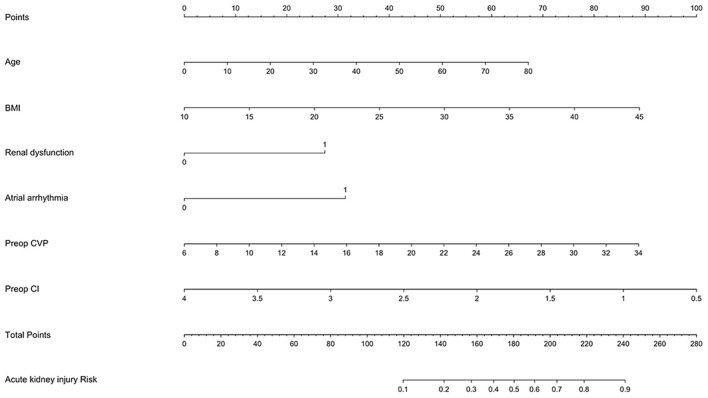
A nomogram of the prediction model for acute kidney injury after pericardiectomy.

**Figure 3:**
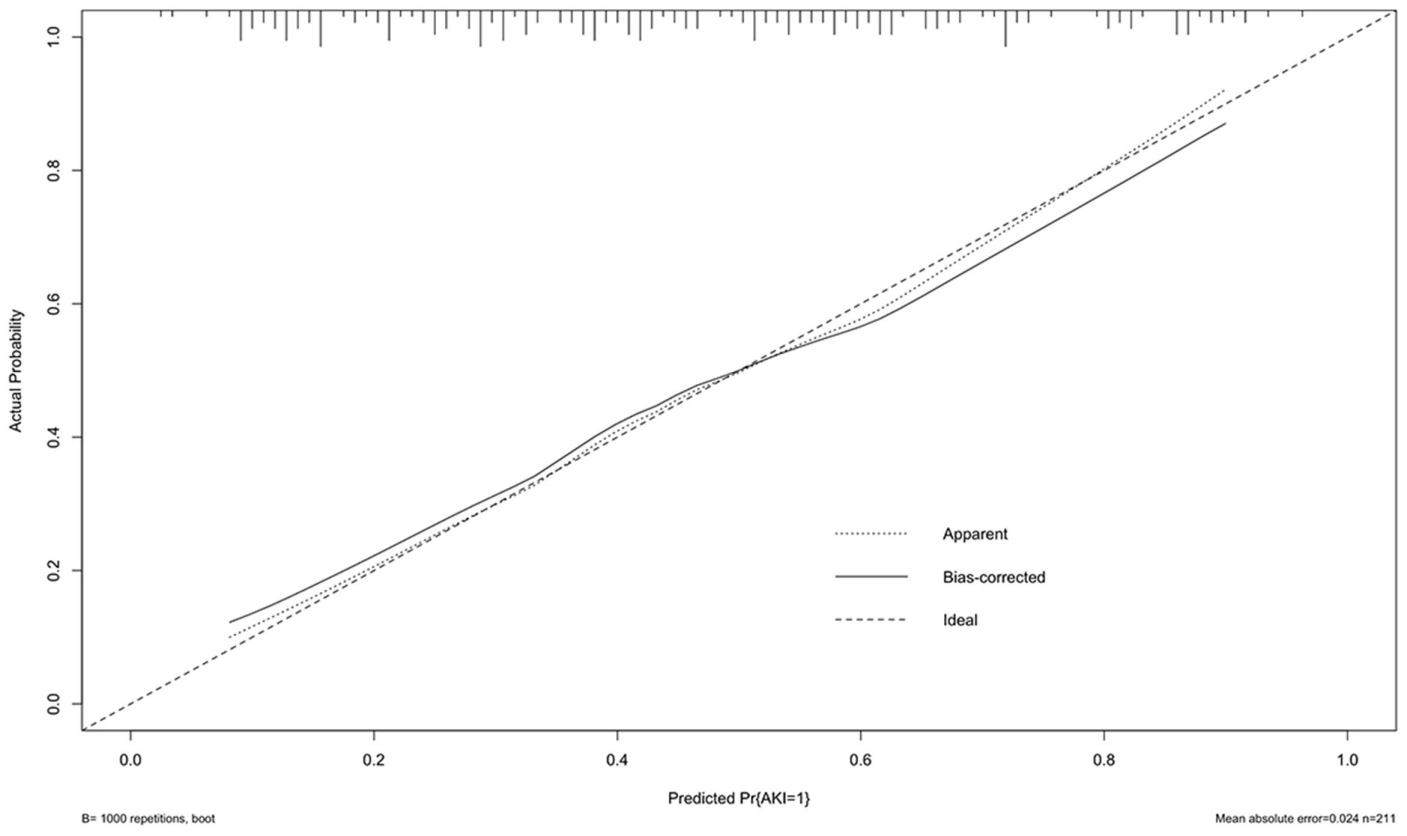
Calibration plot of the prediction model.

**Figure 4:**
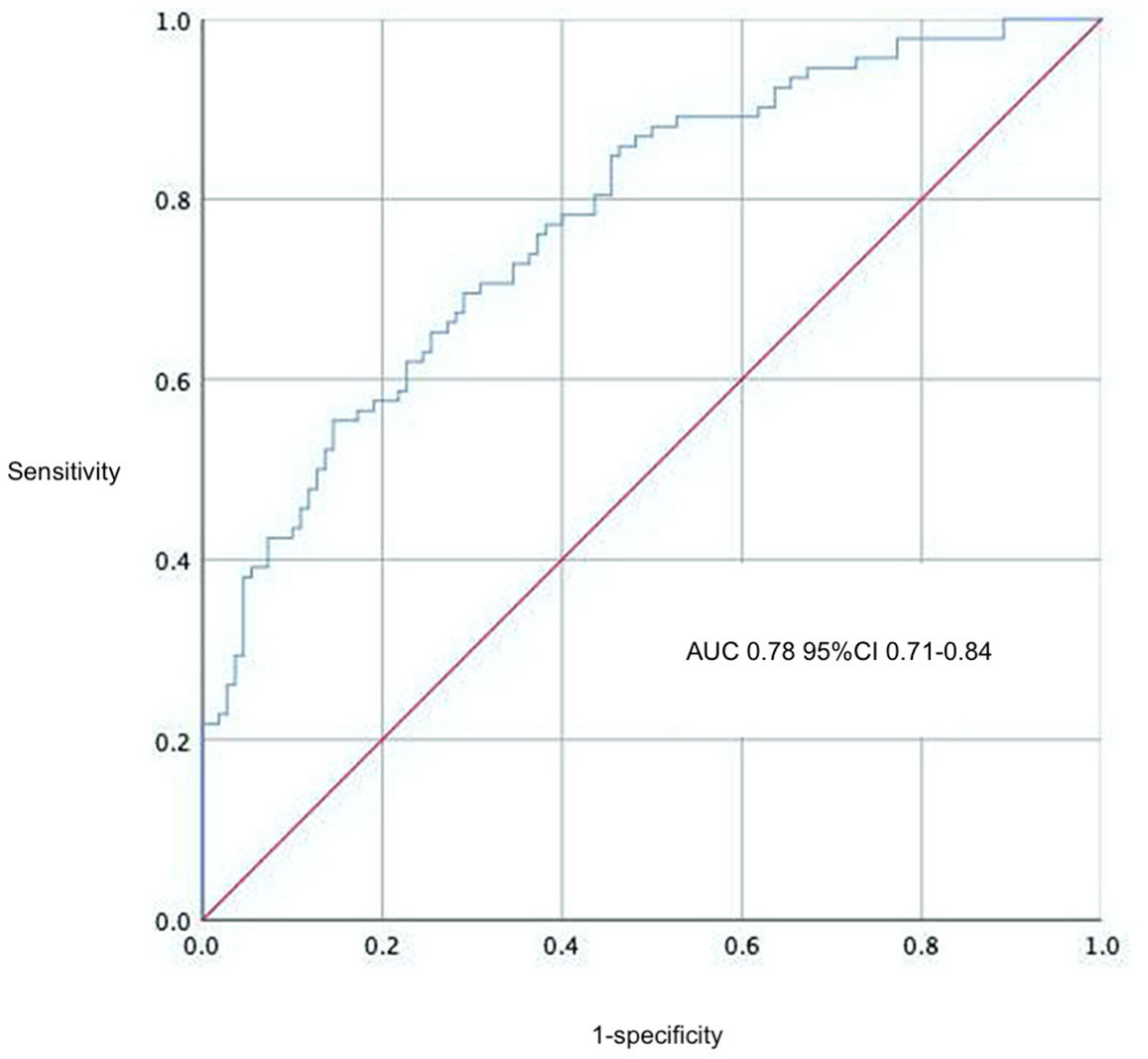
Receiver operator characteristic curve of the prediction model.

A multivariable logistic regression test was performed based on the comparison results between the AKI and non-AKI group to identify predictors for actue kidney injury after pericardiectomy (Table 6). The results showed that advanced age (OR = 1.03, 95% CI 1.01–1.05, *P* = 0.003), high body mass index (OR 1.10, 95% CI 1.01–1.19, *P* = 0.024), preoperative atrial arrhythmia (OR 3.12, 95% CI 1.05–9.30, *P* = 0.041), renal dysfunction (OR 2.70, 95% CI 1.03–7.07, *P* = 0.043), high central venous pressure (OR 1.12, 95% CI 1.04–1.21, *P* = 0.002), and low cardiac index (OR 0.36, 95% CI 0.16–0.77, *P* = 0.009) were independent predictors of acute kidney injury (Figure 1). A nomogram was established based on the regression results (Figure 2). Both the calibration plot (Figure 3) and Hosmer-Lemeshow test (*P* = 0.881) results showed good model fitness. The receiver operating curve depicted an area under the curve value of 0.78 (95% CI: 0.71–0.84, *P* < 0.001) (Figure 4). No regression analysis was performed for mortality or postoperative chronic renal dysfunction due to its low occurrence rate.

**Table 6:** Multivariate analysis of factors predisposing acute kidney injury after pericardiectomy for constrictive pericarditis.

	**B**	**S.E**.	**Exp (B) (95%CI)**	***P***
Age	0.03	0.01	1.03 (1.01, 1.05)	0.003^*^
Body mass index (kg∙m^−2^)	0.09	0.04	1.10 (1.01, 1.19)	0.024^*^
Renal dysfunction	0.99	0.49	2.70 (1.03, 7.07)	0.043^*^
Atrial arrhythmia	1.14	0.56	3.12 (1.05, 9.30)	0.041^*^
Central venous pressure	0.12	0.04	1.12 (1.04, 1.21)	0.002^*^
Cardiac index	−1.03	0.40	0.36 (0.16, 0.77)	0.009^*^

* *Significant difference*.

## Discussion

### Key Results

This study showed that acute kidney injury is a common complication after pericardiectomy for patients with constrictive pericarditis, with an overall incidence of 45.0%, and the rates of hemofiltration and chronic renal dysfunction were 13.7 and 5.2%, respectively. The established prediction model showed that advanced age, high body mass index, renal dysfunction, atrial arrhythmia, high central venous pressure, and low cardiac index were independent predictors of acute kidney injury after pericardiectomy.

### Interpretation

Multiple studies have reported the negative impact of renal dysfunction on patient outcomes and medical costs in pericardiectomy (4, 5, 16, 17), but the predictors of renal dysfunction have hardly been investigated. Huang et al. studied the clinical features of ninety-two patients with constrictive pericarditis, and reported that ventilator time, postoperative creatinine levels and blood transfusion were associated with acute kidney injury after pericardiectomy, with no preoperative factors involved. Additionally, the small sample size significantly violates the statistical strength of their analysis (18). To the best of our knowledge, we failed to identify any study on building a prediction model for acute kidney inury afte pericardiectomy. Studies on other patient populations and surgeries have suggested that critical illness, cardiac or major non-cardiac surgery, nephrotoxic agents, dehydration, advanced age, female sex, black race, chronic disease, diabetes mellitus, cancer, and anemia are associated with postoperative acute kidney injury (11). The underlying mechanisms were considered to involve hypoperfusion, ischemia-reperfusion injury, neurohumoral activation, inflammation, oxidative stress, nephrotoxins, and mechanical factors (19).

Our study demonstrated that patients with constrictive pericarditis were at high risk of developing prerenal insufficiency, and the nomogram provided a precise risk evaluation score for each predictor. One major mechanism for prerenal insufficiency is compromised renal perfusion due to a high central venous pressure and a low cardiac index. Renal perfusion, defined as the mean arterial pressure minus renal venous pressure, is associated with the progression of acute kidney injury in critically ill patients (20, 21). A low cardiac index may reduce mean arterial pressure and renal plasma flow. Anand et al. studied the changes in hormones and fluid in sixteen patients with constrictive pericarditis and found that their renal plasma flow decreased by 49% (22), which predisposed these patients to compromised renal perfusion. Increased central venous pressure can also transmit backward and increase renal venous pressure, thus compromising renal perfusion. A study on patients with a broad spectrum of cardiovascular diseases, including coronary artery, valves and heart rhythm dysfunctions, suggested that central venous pressure and renal function interact with each other, and increased central venous pressure was independently associated with all-cause mortality (23). The study by Lopez et al. on patients receiving on-pump cardiac surgery found that a 60 mmHg min increase above the median venous congestion AUC during the surgery was associated with increased rates of postoperative acute kidney injury (24). Potential mechanisms involved include increased intravascular volume, vascular resistance, and intrathoracic pressure (25). Therefore, preoperative use of inotropes and diuretics was of great importance in this group of patients. The time and doses of diuretics should be carefully planned to reduce preload as much as possible, while not too quick to repeatedly induce transient intravascular depletion (26).

Cardiac and renal function always closely interact with each other (27, 28). In our study population, 46.3% of the AKI group developed low cardiac output, and 81.5% of the patients with low cardiac output also had acute kidney injury. According to a previous study, the total body water of patients with constrictive pericarditis increased by 36% and primarily occurred in the extracellular space (81%) (22). The underlying mechanism was considered as diastolic filling dysfunction-induced hormone disturbances, including impaired secretion of atrial natriuretic factor and stimulation of the renin-angiotensin-aldosterone system (29, 30). After pericardiectomy, the diastolic filling pressure decreased, and a dramatic fluid return from the extravascular space into the intravascular space occurred. Compromised renal function might further aggravate fluid retention and increase preload; if the cardiac function is too poor to adapt to the preload increase, low cardiac output occurrs (1, 10, 31). A rapidly reduced cardiac output also had a negative impact on renal perfusion, which further aggravated kidney injury. Therefore, fluid management is of great importance in the early postoperative time (32), and hemofiltration may be considered for early use in high-risk patients.

## Limitation

This study had some limitations. First, this is a single-center observational study and suffers from all of the shortcomings of this type of study. Second, the study results only applied to patients with constrictive pericarditis receiving isolated pericardiectomy. Patients receiving combined surgeries may have completely different clinical pictures, and their management requires further investigation. Third, the prediction model lacks external validation, and further studies in other medical centers are required to test the model's performance.

## Conclusion

Acute kidney injury is a common complication after pericardiectomy for constrictive pericarditis; if not treated promptly, it may lead to worse outcomes and high medical costs. Patients with advanced age, high body mass index, preoperative renal dysfunction, arrhythmia, high central venous pressure, and low cardiac index were associated with a high risk of postoperative acute kidney injury; therefore, clinicians should make an effort to optimize these factors. A prediction model was established to help clinicians in the early evaluation and management of this complication.

## Data Availability Statement

The raw data supporting the conclusions of this article will be made available by the authors, without undue reservation.

## Ethics Statement

The studies involving human participants were reviewed and approved by Peking Union Medical College Hospital. Written informed consent for participation was not required for this study in accordance with the national legislation and the institutional requirements.

## Author Contributions

JW and CY designed the study. JW collected the data and drafted the manuscript. JW and YZ analyzed the data. YH revised the manuscript. All authors contributed to the article and approved the submitted version.

## Funding

This research was funded by the Beien fundings from the Bethune foundation (Grant number: bnmr-2021-009).

## Conflict of Interest

The authors declare that the research was conducted in the absence of any commercial or financial relationships that could be construed as a potential conflict of interest.

## Publisher's Note

All claims expressed in this article are solely those of the authors and do not necessarily represent those of their affiliated organizations, or those of the publisher, the editors and the reviewers. Any product that may be evaluated in this article, or claim that may be made by its manufacturer, is not guaranteed or endorsed by the publisher.
